# Associations between dietary patterns and 10-year cardiovascular disease risk score levels among Chinese coal miners——a cross-sectional study

**DOI:** 10.1186/s12889-019-8070-9

**Published:** 2019-12-19

**Authors:** Qian Sun, Jin-Sha Ma, Hui Wang, Shu-Hong Xu, Jun-Kang Zhao, Qian Gao, Jian-Jun Huang, Tong Wang

**Affiliations:** 1 Department of Health Statistics, School of Public Health, Shanxi Medical University, 56 Xinjiannanlu Street, Taiyuan, 030001 China; 2 Shaoxing Center for Diseases Prevention and Control, Shaoxing City, 312000 Zhejiang Province China; 3 Department of Neurosurgery, General Hospital of Datong Coal Mining Group, Datong, 037000 China

**Keywords:** Dietary pattern, 10-year atherosclerosis cardiovascular disease risk score level, 10-year ischemic cardiovascular diseases risk score level

## Abstract

**Background:**

Diet-related cardiovascular diseases have produced a large health burden in China. Coal miners are a high-risk population for cardiovascular disease, but there is little evidence concerning associations between coal miners’ dietary patterns and their 10-year cardiovascular disease risk score levels.

**Methods:**

The study included 2632 participants and focused on dietary patterns associated with higher 10-year cardiovascular disease risk score levels. A valid semi-quantitative food frequency questionnaire was used to collect data regarding dietary intake, and dietary patterns were identified using factor analysis combined with cluster analysis. Logistic regression was used to assess associations between dietary patterns and 10-year cardiovascular disease risk score levels.

**Results:**

For ground workers, compared with the ‘Healthy’ pattern, the ‘High-salt’ and ‘Refined grains’ patterns were significantly associated with higher 10-year atherosclerotic cardiovascular disease risk score level (OR: 1.50, 95% CI: 1.02–2.21; OR: 1.92, 95% CI: 1.26–2.93) and 10-year ischemic cardiovascular disease risk score level (OR: 2.18, 95% CI: 1.25–3.80; OR: 2.64, 95% CI: 1.48–4.72) adjusted for gender, and behavioural and socioeconomic factors. The ‘High-fat and salt’ pattern was significantly associated with higher 10-year ischemic cardiovascular disease risk score level (OR: 1.97, 95% CI: 1.13–3.42). For underground workers, the ‘High-salt’ pattern was significantly associated with higher 10-year atherosclerotic cardiovascular disease risk score level (OR: 1.65, 95% CI: 1.16–2.36) and 10-year ischemic cardiovascular disease risk score level (OR: 1.76, 95% CI: 1.09–2.84).

**Conclusions:**

This study provides evidence for dietary patterns associated with higher 10-year cardiovascular disease risk score levels in Chinese miners, and facilitates relevant departments in designing effective dietary guidelines to ameliorate dietary structures.

## Background

Currently, cardiovascular disease (CVD) accounts for about one-third of all deaths worldwide [1]. In China, CVD is the leading cause of death and disease burden [2–4], and two out of five deaths are attributed to CVD [3]. The mortality, incidence, and prevalence of diet-related CVD have been increasing over the past 30 years, and now the number of CVD patients has reached 290 million [3]. Among CVDs, atherosclerosis cardiovascular diseases (ASCVD) have remained at a high prevalence, and ischemic cardiovascular diseases (ICVD) have produced large health burden [2, 5]. Each CVD is undesirable for maintaining a healthy population, and previous studies have shown that coal mining areas have higher CVD risk and mortality than non-coal mining areas [6–8], so to improve the primary prevention of CVD, coal miners’ health should be taken seriously.

In recent years, the global food system and food supply have been shifting rapidly [9]. The Chinese diet used to be characterized by coarse grains, but now it has been transformed into a dietary structure based on refined carbohydrates [10]. At the same time, because of improvements in living conditions, consumption of meat in the diet has increased significantly [11, 12]. Dietary habits play a key role in CVD primordial prevention [13], and a diet high in fat or refined foods is clearly not conducive to the control or prevention of CVD [14]. Coal miners are characterized by lower socioeconomic status and education levels compared to the general population, and for these reasons, coal miners are more likely to have poor eating habits [15]. Therefore, it is necessary to pay attention to the suboptimal diet of coal miners for CVD prevention. Current evidence on the impact of coal miners’ dietary pattern on CVD is scant. Therefore, this study focused on the dietary patterns associated with 10-year CVD risk score levels, so it may offer potential benefits for primary prevention of CVD in miners by identifying suboptimal diets, and facilitate relevant departments in designing effective dietary guidelines.

Considering the potential influence of work environment [16, 17], this study stratified the participants based on workplace and identified the dietary patterns associated with higher 10-year CVD risk score levels, including 10-year ASCVD and ICVD risk score levels, separately.

## Methods

### Study population

**Fig. 1.**
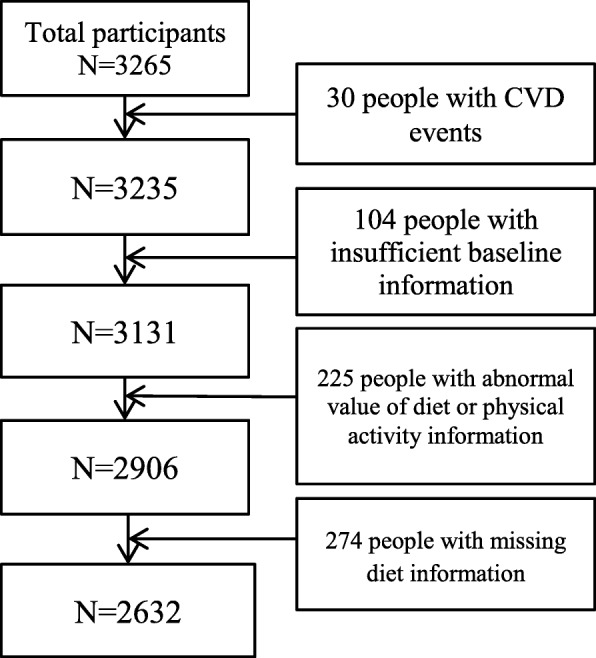
Flowchart of participants through the study

Participants were drawn from The TONGMEI study, which was designed to investigate coal miners’ health status and was conducted in 2013 in Shanxi Province in China. This study enrolled 3265 people aged between 35 and 65 years old. Of these, 30 people with self-reported cardiovascular events, 104 people with insufficient data about baseline information, 274 people with missing data about one or more variables of diet required in the analysis, and 225 people with abnormal values of diet or physical activity information identified by relative guidelines, were excluded from the analysis [18]. Therefore, the current analyses were based on data from 2632 people. The sampling of the TONGMEI study, a cross-sectional study, has been described in detail elsewhere [19]. A flowchart presents the analytical sample, excluding ineligible individuals (Fig. 1).

### Assessment of general information and covariates

Data on general information including marital status, education level, monthly income, work type, alcohol consumption, and family history of illness were collected using a self-administered baseline questionnaire. In addition, the physical activity level was assessed by the international physical activity questionnaire including work-related activity, housework activity, leisure, transport-related activity, exercise, sitting posture, and sleeping time. Details on the collection of blood samples and measurement of anthropometrics indicators have been previously published [19]. The blood pressure (BP) of participants was categorized into Normal (systolic BP (SBP) < 120 mmHg and diastolic BP (DBP) < 80 mmHg), Elevated (SBP ≥ 120 mmHg or DBP ≥ 80 mmHg), and Hypertension (SBP ≥ 140 mmHg or DBP ≥ 90 mm) [20]. Body mass index (BMI) was computed as weight divided by the square of height. Alcohol users were defined as those consuming alcohol at least once per month in the past year [21]. Smokers were defined as those smoking at least one cigarette per day during the past month [22]. The workplace of miners was divided into ground and underground; ground workers engaged in coal transportation, operation management, communication, power supply, and some office jobs. Underground workers mainly engaged in machinery driving, reserves, and some auxiliary jobs.

### Cardiovascular risk score levels

The 10-year CVD risk scores include 10-year ICVD and ASCVD risk scores, which are effective tools with good performance for 10-year CVD risk prediction among the Chinese population [23]. Meanwhile, 10-year CVD risk scores were recommended for application in CVD screening by the Chinese Guidelines for the Prevention of Cardiovascular Diseases [24, 25]. The 10-year CVD risk scores were calculated by the specific risk evaluation models, further converted into absolute risk (%), and graded into risk levels according to the relevant guidelines. The 10-year ASCVD risk was defined as the probability of developing the first ASCVD event over a 10-year period among people free from ASCVD at the beginning [26]. The 10-year ASCVD risk score was calculated by the sex-specific evaluation figure of ASCVD risk developed by the Joint Committee of Dyslipidemia Management [27]; the risk factors included in evaluation models were age, BP, total cholesterol (TC), low-density lipoprotein cholesterol, high-density lipoprotein cholesterol, current smoking status, and diabetes. In additions, four risk levels were determined according to the guideline: Low risk (< 5%), Medium risk (5–9%), High risk (10–19%), and Extremely high risk (≥20%) [27].

The 10-year ICVD risk was defined as the probability of developing the first ICVD event over a 10-year period among people free from ICVD at the beginning [24]. The 10-year ICVD risk score was calculated using a sex-specific evaluation sheet published by the Chinese Society of Cardiology of the Chinese Medical Association [24], and updated based on a previous study [28]; the risk factors included in evaluation models were age, SBP, BMI, TC, current smoking status, and diabetes. Additionally, the following three risk levels were determined: Extremely low risk (≤5%), Low risk (5–10%), and Medium-high risk (≥10%) [24].

### Dietary assessment

Dietary data were collected from the semi-quantitative FFQ [19]. All of the risk factors and FFQ were collected in the same year. To minimize participants’ recall bias as much as possible, this study made a commitment to provide each participant with a health report to increase their motivation to answer accurately, and provided uniform metrics of food consumption and different portions of foods for reference by participants at the survey site. Food items consumed frequently in the population were categorized into 20 categories including rice, wheat flour, cereal, tubers(e.g. white potatoes), fried dough, pork, red meat, poultry, viscera, fish and shrimp, dairy products, beans and bean products, egg and egg dishes, vegetables, pickled vegetables, salted and preserved vegetables, vermicelli, pastry, fruits, and nuts [19]. Participants were asked to recall the portion size, frequency, and cycle of 20 food categories for the past year. The traditional Chinese unit Liang was used to measure the portion size, participants were helped to estimate sizes accurately, and finally converted to grams (e.g. 1 Liang = 50 g). Information on the cycle of consumption for each food category was collected by using five categories (never, daily, weekly, monthly, and yearly). Finally, the mean intake in grams/day of each food category was assessed based on portion size, frequency, and cycle.

### Dietary pattern identification

Dietary patterns were derived by an exploratory approach of factor analysis combined with cluster analysis, a method that has been widely used in the construction of dietary patterns [29, 30]. Factor analysis identified food groups that were frequently consumed together. Four factors were retained in ground and underground workers, by combining with the Akaike information criterion value, Schwarz Bayesian criterion value (which produces fewer factors with less significance) [31], scree test, and eigenvalues. Foods with factor loading ≥0.2 were retained after varimax rotation, while foods with factor loading ≥0.35 were considered highly important to each factor [32]. Participants were assigned a factor score computed for detecting the actual underlying and unobservable factor [33]; meanwhile, the factor scores were used in the cluster analysis after being standardized.

The number and cluster seeds of clusters were determined through hierarchical cluster analysis, and the final clusters (patterns) were identified by K-means cluster analysis. All participants were assigned to one of the clusters (patterns), and the descriptive names were assigned for each pattern according to the cluster means of factors scores that contributed relatively highly [34].

### Statistical analyses

This study stratified the participants based on the workplace and identified the dietary patterns, separately. Descriptive statistics were used to examine the association of each risk score level with demographic and lifestyle variables.

The dietary patterns of workers in the two workplaces were generated by factor analysis combined with cluster analysis. Each participant received a factor score by factor analysis implemented in SAS PROC FACTOR, and the factor scores were standardized prior to the cluster analysis. Hierarchical cluster analysis was conducted to identify the most appropriate number and the cluster seeds for the subsequent K-means cluster analysis. Thereafter, the construction of final dietary patterns using K-means cluster analysis performed by SAS PROC FASTCLUS [34].

Logistic regression was used to assess the association between dietary patterns and the 10-year CVD risk score levels. The 10-year CVD risk score level was the dependent variable, the dietary patterns were the independent variables, and dummy variables were calculated in the regression models. We fitted three models by adjusting for potential confounding effects. Model 1 included the dietary pattern. In model 2, besides the primary variable of interest, we further adjusted for gender and drinking status (No/Yes). In model 3, we further adjusted for education level (Bachelor degree or above, Junior college and senior high school, Junior high school or below), monthly income (≤4000, 4000–6000, 6000–8000, ≥8000), marital status (married, others), physical activity level (Inactive, Minimally Active, Health-enhancing physical activity), and family history (No/Yes). Among the three models, we did not adjust for age, smoking status, and blood pressure, because these covariates were included in the outcome measure.

We conducted sensitivity analysis to observe the impact of missing data of dietary intake on dietary patterns, imputed the missing data of dietary intake in the imputed dataset, the maximum-likelihood estimates via EM algorithm were computed for the missing data [35], performed by SAS PROC MI. In sensitivity analysis, we rebuilt dietary patterns in the same way, and examined the stability of dietary patterns. All statistical analyses were performed using SAS 9.4 (SAS Institute, Inc.) and *p* values < 0.05 were considered statistically significant.

## Results

### Characteristics of the sample

The main analyses set was based on 2632 participants aged 35–65 with no CVD events. The descriptive statistics of the demographic factors and their association with the 10-year CVD risk score levels are shown in Table 1. For 10-year ASCVD risk score level, one participant aged 65 was excluded because the 10-year ASCVD risk evaluation model was developed for adults aged 35–64. The remaining participants were classified into four levels: Low risk (*N* = 1753, 66.6%), Medium risk (*N* = 551, 20.9%), High risk (*N* = 259, 9.8%), and Extremely high risk (*N* = 68, 2.6%). For 10-year ICVD risk score level, participants were classified into three levels: Extremely low risk (*N* = 2222, 84.4%), Low risk (*N* = 326, 12.4%), and Medium-high risk (*N* = 84, 3.2%).

**Table 1:** The descriptive statistics of demographic factors and their association with 10-year CVD risk score levels^a^

Demographic Factors	10-year ICVD risk score level N (%)			χ^2^	P	10-year ASCVD risk score level N (%)				χ^2^	P
	Extremely low risk *N* = 2222(84.4)	Low risk *N* = 326(12.4)	Medium-high risk *N* = 84(3.2)			Low risk *N* = 1753(66.6)	Medium risk *N* = 551(20.9)	High risk *N* = 259(9.8)	Extremely high risk *N* = 68(2.6)		
Age				387.1	<.0001					573.9	<.0001
35–44	1125(50.6)	23(7.1)	6(7.1)			989(56.4)	125(22.7)	31(11.9)	9(13.1)		
45–54	934(42.0)	196(60.1)	46(54.8)			717(40.9)	261(47.4)	160(61.8)	38(55.9)		
≥55	163(7.4)	107(32.8)	32(38.1)			47(2.7)	165(29.9)	68(26.3)	21(30.9)		
Gender				49.4	<.0001					133.2	<.0001
male	1853(83.4)	312(95.7)	84(100)			1401(79.9)	521(94.6)	258(99.6)	68(100.0)		
female	369(16.6)	14(4.3)				352(20.1)	30(5.4)	1(0.4)	0(0.0)		
Marital status				2.3	0.31					6.9	0.07
married	2168(97.6)	322(98.8)	83(98.8)			1705(97.3)	545(98.9)	254(98.1)	68(100.0)		
others	54(2.4)	4(1.2)	1(1.2)			48(2.7)	6(1.1)	5(1.9)	0(0.0)		
Educational level				40.5	<.0001					36.9	<.0001
Bachelor degree or above	194(8.7)	8(2.5)	3(3.6)			160(9.1)	34(6.2)	8(3.1)	3(4.4)		
Junior college and senior high school	1310(58.9)	174(53.4)	37(44.1)			1041(59.4)	309(56.1)	143(55.2)	28(41.2)		
Junior high school or below	718(32.3)	144(44.2)	44(52.4)			552(31.5)	208(37.8)	108(41.7)	37(54.4)		
Monthly income (RMB)				13.1	0.0416					19.4	0.0220
≤4000	561(25.3)	104(31.9)	25(29.8)			442(25.2)	150(27.2)	80(30.9)	17(25.0)		
4000–6000	959(43.2)	147(45.1)	37(44.1)			754(43.0)	234(42.5)	129(49.8)	26(38.2)		
6000–8000	509(22.9)	54(16.6)	17(20.2)			409(23.3)	119(21.6)	35(13.5)	17(25.0)		
≥8000	193(8.7)	21(6.4)	5(5.9)			148(8.4)	48(8.7)	15(5.8)	8(11.8)		
Smoke				94.0	<.0001					113.0	<.0001
No	1027(46.2)	68(20.9)	16(19.1)			856(48.8)	182(33.0)	69(26.6)	4(5.9)		
yes	1195(53.8)	258(79.1)	68(80.9)			897(51.2)	369(66.9)	190(73.4)	64(94.1)		
Drink				46.4	<.0001					67.9	<.0001
No	1346(60.6)	152(46.6)	27(32.1)			1105(63.0)	284(51.5)	113(43.6)	22(32.4)		
Yes	876(39.4)	174(53.4)	57(67.9)			648(36.9)	267(48.5)	146(56.4)	46(67.7)		
BMI(kg/m2)				87.2	<.0001					61.1	<.0001
≤23	688(30.9)	49(15.0)	12(14.3)			565(32.2)	122(22.1)	45(17.4)	17(25)		
23–27.5	1111(50.0)	158(48.5)	37(44.1)			865(49.3)	277(50.3)	136(52.5)	27(39.7)		
> 27.5	423(19.0)	119(36.5)	35(41.7)			323(18.4)	152(27.6)	78(30.1)	24(35.3)		
BP				553.3	<.0001					1321.8	<.0001
Normal	715(32.2)	0(0.0)	0(0.0)			658(37.5)	55(9.9)	1(0.4)	0(0.0)		
Elevated	964(43.4)	73(22.4)	0(0.0)			906(51.7)	131(23.8)	0(0.0)	0(0.0)		
Hypertension	543(24.4)	253(77.6)	84(100.0)			189(10.8)	365(66.2)	258(99.6)	68(100.0)		
Workplace				1.9	0.17					4.4	0.22
Ground	1024(46.1)	161(49.4)	43(51.2)			803(45.8)	278(50.5)	117(45.2)	29(42.7)		
Underground	1198(53.9)	165(50.6)	41(48.8)			950(54.2)	273(49.6)	142(54.8)	39(57.4)		
Physical activity level				7.4	0.12					9.1	0.16
Inactive	36(1.6)	12(3.7)	2(2.4)			26(1.5)	14(2.5)	8(3.1)	2(2.9)		
Minimally Active	672(30.2)	100(30.7)	22(26.2)			520(29.7)	169(30.7)	88(33.9)	16(23.5)		
Health-enhancing physical activity	1514(68.1)	214(65.6)	60(71.4)			1207(68.9)	368(66.8)	163(62.9)	50(73.)		
Family history				4.3	0.12					3.2	0.37
No	1312(59.1)	183(56.1)	41(48.8)			1031(58.8)	324(58.8)	147(56.8)	33(48.5)		
Yes	910(40.9)	143(43.9)	43(51.2)			722(41.2)	227(41.2)	112(43.2)	35(51.5)		

^*a*^*CVD* cardiovascular diseases, *ASCVD* atherosclerosis cardiovascular diseases, *ICVD* ischemic cardiovascular disease

### Dietary patterns

The factor loadings derived from factor analysis are presented in Table 2. Among the ground workers, four factors were derived, explaining 36% of the total variance in consumption of the foods. The first factor included red meat, poultry, viscera, fish and shrimp, pork, fried dough, and pastry. The second factor included tubers, wheat flour, beans and bean products, vegetables, pastry, vermicelli, cereal, and eggs and egg dishes. The third factor included fried dough, salted and preserved vegetables, pickled vegetables, and vermicelli. The fourth factor included fish and shrimp, beans and bean products, vegetables, fruits, dairy products, nuts, rice, cereal, and eggs and egg dishes.

**Table 2:** Factors and factor loadings derived from FFQ among miners^a^

Foods	Ground worker				Underground worker			
	Factor 1:Meat, Fried food	Factor 2:Refined grain, Tubers	Factor3: salt, processed vegetables	Factor 4: Cereal, Fruits	Factor1: Red meat, Viscera	Factor2: Beans, Vegetables, Tubers	Factor3: Cereal, Fruits, Dairy products	Factor4: salt, processed vegetables
Red meat	0.71				0.69			
Poultry	0.64				0.56			
Viscera	0.59				0.59	0.25		
Fish and shrimp	0.58			0.31	0.58		0.26	
Pork	0.46				0.45			
Fried dough	0.36		0.34		0.32	0.35		
Tubers		0.59				0.43	0.26	
Wheat Flour		0.57				0.35		
Beans and bean products		0.56		0.20		0.59		
Vegetables		0.55		0.20		0.57		
Pastry	0.24	0.42				0.45		
Salted and preserved vegetables			0.81					0.83
Pickled vegetables			0.78					0.82
Vermicelli		0.29	0.35			0.47		
Fruits				0.68			0.50	
Dairy products				0.52			0.50	
Nuts				0.45	0.22	0.34		
Rice				0.37			0.53	
Cereal		0.29		0.35			0.57	
Eggs and egg dishes		0.27		0.32	0.23	0.24	0.26	
Eigen value	2.65	1.80	1.50	1.25	2.52	1.67	1.39	1.29

^a^Only factor loadings ≥0.2 are presented. The factor loadings ≥0.35 are underlined

Participants were given a factor score after factor analysis, and assigned to each of the patterns after the cluster analysis. The final dietary patterns were identified by K-means cluster analysis, and are presented in Table 3. Each pattern was labeled according to the cluster means of factor scores, which were relatively high; the high mean factor scores of each pattern are underlined in Table 3. Among the ground workers, the first pattern with a high mean factor score was on the fourth factor, characterized by high intakes of fruits, dairy products, nuts, rice, cereal, eggs and egg dishes, and called ‘Healthy’. A pattern with a high mean factor score on the third factor, characterized by high intake of salted and preserved vegetables, pickled vegetables, and vermicelli was called ‘High-salt’. The other cluster patterns, respectively, were named ‘High-fat and salt’ and ‘Refined grains’ in the same way.

**Table 3:** Cluster means of factor scores according to clusters for miners^a^

Dietary pattern	Factors			
	Factor1	Factor2	Factor3	Factor4
Ground worker				
Healthy	−0.13	− 0.49	−1.11	0.61
High-fat and salt	0.97	−0.06	0.54	0.16
High-salt	−0.82	0.17	0.64	0.14
Refined grains	0.03	0.43	−0.68	−1.36
Underground worker				
Healthy	−0.13	0.01	0.43	−1.27
Northern	−0.79	0.45	0.04	0.58
High-fat	0.96	0.27	−0.02	0.29
High-salt	−0.19	−1.48	−0.72	0.22

^a^The high mean factor scores of each pattern are underlined

Among the underground workers, four factors were derived, explaining 34% of the total variance in the consumption of the foods (Table 2). Dietary pattern included four patterns, namely ‘Healthy’, ‘High-salt’, ‘High-fat’ and ‘Northern’ patterns (Table 3). Compared to the ‘Healthy’ pattern, the ‘High-salt’ pattern had a high score on the fourth factor and primarily consisted of salted and preserved vegetables and pickled vegetables. The other patterns, were named ‘High-fat’ and ‘Northern’ in the same way.

In both ground and underground workers, dietary patterns included four sets of patterns. In the following regression analysis, the dietary patterns were the independent variables and the dummy variables were calculated. Compared to the other patterns, the ‘Healthy’ pattern primarily consisted of healthier foods, and served as the reference group for exploring the association of three other suboptimal patterns with 10-year CVD risk score levels.

### Dietary patterns and distributions of sample characteristics

Participants with a ‘High-salt’ pattern were the major group and constituted 31.9% of the ground workers (Additional file 1: Table S1). Meanwhile, the local special suboptimal pattern was characterized by the ‘High-salt’pattern, which was also identified in the underground workers. In addition, participants with a ‘High-fat’ pattern were the major group, and constituted 31.1% of the underground workers (Additional file 1: Table S1).

### Dietary patterns and 10-year CVD risk score levels

For all logistic regression analyses, the ‘Healthy’ patterns were served as the reference group both in ground and underground workers. After adjusting for all covariates, among the ground workers, the ‘High-salt’ pattern (OR: 1.50; 95% CI: 1.02–2.21) and ‘Refined grains’ pattern (OR: 1.92; 95% CI: 1.26–2.93) were significantly associated with higher 10-year ASCVD risk score levels after adjusting for all covariates. Among the underground workers, the ‘High-salt’ pattern was significantly associated with higher 10-year ASCVD risk score levels (OR: 1.65; 95% CI: 1.16–2.36) (Table 4), and the association was still statistically significant in sensitivity analyses (Additional file 1: Table S2).

**Table 4:** Associations between dietary patterns and 10-year ASCVD risk score level for miners^a^

ICVD risk score level	Ground workers *N* = 1227				*P* value	Underground workers *N* = 1404				*P* value
	Healthy pattern	High-fat and salt pattern	High-salt pattern	Refined grains pattern		Healthy pattern	Northern Pattern	High-fat pattern	High-salt pattern	
N (%)	273(22.2)	359 (29.2)	392(31.9)	203(16.5)		333(23.7)	421(29.9)	437(31.1)	213(15.2)	
Model 1	1.00	2.52 (1.76–3.59) *P* < .0001	1.76 (1.23–2.52) *P* = 0.0020	3.69 (2.49–5.47) *P* < .0001	<.0001	1.00	1.15 (0.85–1.57) *P* = 0.359	1.03 (0.76–1.39) *P* = 0.865	1.74 (1.23–2.48) *P* = 0.0019	0.0050
Model 2	1.00	1.48 (1.01–2.16) *P* = 0.0448	1.64 (1.12–2.40) *P* = 0.0113	2.17 (1.44–3.28) *P* = 0.0002	0.0016	1.00	1.17 (0.86–1.59) *P* = 0.315	0.98 (0.72–1.33) *P* = 0.888	1.68 (1.18–2.38) *P* = 0.0040	0.0066
Model 3	1.00	1.29 (0.88–1.91) *P* = 0.188	1.50 (1.02–2.21) *P* = 0.0399	1.92 (1.26–2.93) *P* = 0.0023	0.0168	1.00	1.05 (0.77–1.44) *P* = 0.762	0.98 (0.72–1.34) *P* = 0.896	1.65 (1.16–2.36) *P* = 0.0057	0.0086

^a^*ASCVD* atherosclerosis cardiovascular diseases.; Model 1 included the dietary pattern. Model 2 adjusted for gender and drinking status. Model 3 adds education level, monthly income, BMI, marital status, physical activity level and family history to the above

Among the ground workers, compared with the ‘Healthy’ pattern, the ‘High-fat and salt’ pattern (OR: 1.97; 95% CI: 1.13–3.42), ‘High-salt’ pattern (OR: 2.18; 95% CI: 1.25–3.80), and ‘Refined grains’ pattern (OR: 2.64; 95% CI: 1.48–4.72) were significantly associated with higher 10-year ICVD risk score levels. Among the underground workers, the ‘High-salt’ pattern was significantly associated with higher 10-year ICVD risk score levels (OR: 1.76; 95% CI: 1.09–2.84), whereas neither the ‘Northern’ (OR: 1.21; 95% CI: 0.78–1.85) and ‘High-fat’ patterns (OR: 1.11; 95% CI: 0.72–1.73) showed a significant association with 10-year ICVD risk score levels in the adjusted analysis (Table 5). In sensitivity analysis, nearly all dietary patterns were still significantly associated with higher 10-year ICVD risk score levels, except for the ‘High-fat and salt’ pattern (Additional file 1: Table S3).

**Table 5:** Associations between patterns and 10-year ICVD risk score level for miners^a^

ICVD risk score level	Ground workers *N* = 1228				*P* value	Underground workers *N* = 1404				*P* value
	Healthy pattern	High-fat and salt pattern	High-salt pattern	Refined grains pattern		Healthy pattern	Northern Pattern	High-fat pattern	High-salt pattern	
N (%)	273(22.2)	359 (29.2)	392(31.9)	204(16.6)		333(23.7)	421(29.9)	437(31.1)	213(15.2)	
Model 1	1.00	3.24 (1.91–5.53) *P* < .0001	2.44 (1.42–4.18) *P* = 0.0012	4.72 (2.69–8.25) *P* < .0001	<.0001	1.00	1.34 (0.88–2.05) *P* = 0.173	1.13 (0.74–1.74) *P* = 0.572	1.89 (1.18–3.02) *P* = 0.0079	0.0356
Model 2	1.00	2.06 (1.19–3.56) *P* = 0.0097	2.33 (1.34–4.05) *P* = 0.0027	2.97 (1.68–5.28) *P* = 0.0002	0.0013	1.00	1.37 (0.89–2.09) *P* = 0.147	1.06 (0.69–1.64) *P* = 0.781	1.79 (1.12–2.87) *P* = 0.0155	0.0426
Model 3	1.00	1.97 (1.13–3.42) *P* = 0.0162	2.18 (1.25–3.80) *P* = 0.0062	2.64 (1.48–4.72) *P* = 0.0010	0.0113	1.00	1.21 (0.78–1.85) *P* = 0.394	1.11 (0.72–1.73) *P* = 0.637	1.76 (1.09–2.84) *P* = 0.0202	0.0881

^a^*ICVD*, ischemic cardiovascular disease.; Model 1 included the dietary pattern. Model 2 adjusted for gender and drinking status. Model 3 adds education level, monthly income, marital status, physical activity level and family history to the above

## Discussion

In previous studies, more attention was paid to coal miners’ occupational injuries and other poor health status [36, 37], but little evidence concerning coal miners’ CVDs has been reported. Previous research showed that coal mining areas are associated with poorer health status and higher incidence of CVD [38, 39], so primary prevention for coal miners should be taken seriously. Dietary habits are recognized as a key modifiable factor in CVD prevention [40]. Given the complexity of diet, there may be stronger effects on health of overall diet than any single component [40]. Therefore, research focused on single foods or nutrients related to coal miners’ health is not enough [41, 42], and it is important to study not only individual foods or nutrients, but also the effects of the entire diet [14].

This study focused on the whole dietary pattern associated with 10-year CVD risk score levels and presented different dietary patterns in ground and underground workers, the dietary differences may due to social networks or work pressure [43]. The majority of coal miners in this study had extremely low and low cardiovascular risk score levels, but low risk does not mean no risk. To prevent CVD risk from developing from low to high, more attention should be given to CVD prevention.

Three suboptimal diets associated with higher 10-year CVD risk score levels were identified in this study. The local special suboptimal pattern was ‘High-salt’ characterized by salted and preserved vegetables and pickled vegetables. A positive association was found between the ‘High-salt’ pattern and 10-year ASCVD risk score levels both in ground and underground workers, and the odds ratios were 1.50 (95% CI: 1.02–2.21) and 1.65 (95% CI: 1.16–2.36), respectively. The ‘High-salt’ pattern was also associated with higher 10-year ICVD risk score levels. This finding is consistent with evidence that a salt-rich diet is related to cardiovascular events in non-coal mining areas [44, 45]. However, there are differences in the food combinations of diets in different populations. Compared with salt-rich diets in Western countries, the ‘High-salt’ pattern identified in this study was mainly characterized by salted and preserved vegetables and pickled vegetables, not manufactured foods [46]. A high salt intake is not conducive to blood pressure control, and is positively related to CVD [44], which is consistent with our finding. Because of the sociocultural factors, a ‘High-salt’ pattern remains pervasive in Chinese diets, and was also identified in the Henan Rural Cohort Study of China [47]. Although dietary salt intake has shown a downward trend in recent years, it is still more than twice the recommended amount (China: < 6 g/day, WHO: < 5 g/day) [3]. Given Chinese taste preferences, specific strategies should be designed to improve their dietary structure to prevent CVD, alternative techniques of food cooking should be advanced instead of pickling, and it is essential to find alternatives to replace salt in cooking, reduce dietary salt intake, and retain the nutrients in foods [3, 48].

Chinese traditional diets used to be dominated by coarse food grains (e.g. maize, sorghum, etc.), and now are loaded highly with refined grains (e.g. white rice, wheat flour, etc.) [12]. The shift in dietary pattern is clearly detrimental to CVD prevention. Poor-quality carbohydrates are associated with CVD risk [10]. As the results showed, the ‘refined grains’ pattern, characterized by wheat flour, pastry, and other starch-rich foods, was positively associated with 10-year ASCVD (OR: 1.92, 95% CI: 1.26–2.93) and 10-year ICVD risk score level (OR: 2.64, 95% CI: 1.48–4.72). Refined food diets have also been identified in northern of China [49], and even in other Asian countries, refined food diets related to CVD risk have also been reported [50]. Currently, refined foods account for a large portion of global dietary patterns. In the United States, nearly three in four Americans consume too many refined foods, such as white bread and corn flakes instead of white rice and pastry [14, 51]. Refined foods have similar metabolic characteristics as high-starch vegetables (e.g. white potatoes), and can be digested rapidly without a fiber-rich structure [14]. Whether it is white bread in Western diets or pastry in Chinese diets, all of these will produce brisk rises in blood glucose and induce multiple adverse manifestations associated with cardiovascular events [52–54]. Consequently, reducing refined grain consumption in the diet for cardiovascular health are major dietary priorities, based on their adverse effects and prevalence in modern diets [14].

The association between a high-fat diet characterized by high intake of meats and CVD risk is complicated, as the category and ingredients of the meats have effects on CVD risk, such as sodium and heme iron [14]. Fanelli et al. found that ‘Western’ diets along with meats and sandwiches, in African-Americans were associated with higher 10-year ASCVD risk [26]. Among the underground workers, like the ‘Northern’ pattern, the ‘High-fat’ pattern had high factor loadings, not only on meats, but also on vegetables and beans. That may explain why there was no significant association between these diets and 10-year CVD risk score levels. Although there are many controversies, it has been established that certain foods, including processed meats and fried products, are positively associated with CVD risk [55, 56]. Intake of fish and nuts is inversely related to CVD risk [57–59]. Thus, it would be prudent to choose consume more fish and nuts, and avoid fried foods.

Participants in study all had the same demographic characteristics and would be likely to have shared similar genetic characteristics. Suboptimal diets that are positively associated with CVD have diverse characteristics in different occupations and regions [14], so it is important to examine dietary patterns in different populations with various sociocultural backgrounds. This study had two strengths. First, this study focused on CVD prevention, and identified the potentially high-risk group by calculating 10-year CVD risk score, which combined some major adverse cardiovascular events that will occur more frequently than single cardiovascular events [60]. Second, considering the complexity of diet, this study focused on dietary patterns instead of single foods or nutrients, providing evidence that suboptimal dietary patterns are associated with higher CVD risk score levels, conforming to current research trends in public health.

This study has certain limitations. A major limitation of its cross-section design is that causal inferences cannot be made. Thus, the long-term impact of dietary patterns on CVD need to be further confirmed [33]. In addition, over/underestimation due to recall bias may have been possible in this study, as the collection of data regarding food intake was dependent on the participants’ memories and motivation. Finally, there is the inherent subjectivity of both factor and cluster analysis derived patterns in the study.

## Conclusions

In conclusion, this study showed that dietary patterns were associated with 10-year CVD risk score levels, which is consistent with previous studies conducted outside of China. Diet is a major modifiable factor in CVD prevention. This study provides evidence to food-based dietary guidelines that can be used to ameliorate people’s dietary habits. However, further study is also needed to confirm the long-term impact of these dietary patterns.

## Supplementary information

**Additional file 1 : Table S1** Demographic characteristics described based on four patterns. **Table S2**: Associations between patterns and 10-year ASCVD risk score level for miners in imputed dataset. **Table S3**: Associations between patterns and 10-year ICVD risk score level for miners in imputed dataset.

## Data Availability

The data that support the findings of this study are available from the corresponding author upon reasonable request.

## Abbreviations

ASCVD: atherosclerosis cardiovascular disease
BMI: body mass index
BP: blood pressure
CVD: cardiovascular disease
DBP: diastolic blood pressure
FFQ: food frequency questionnaire
ICVD: ischemic cardiovascular disease
KMO: Kaiser-Meyer-Olkin
SBP: systolic blood pressure
TC: total cholesterol
