# Supplementary material for: Associations between dietary patterns and 10-year cardiovascular disease risk score levels among Chinese coal miners——a cross-sectional study
Source: BMC Public Health. 2019 Dec 19;19:1704. doi: 10.1186/s12889-019-8070-9 (PMC6923962; doi:10.1186/s12889-019-8070-9)
Supplement: Supplementary file 1 — Additional file 1 : Table S1 Demographic characteristics described based on four patterns. Table S2: Associations between patterns and 10-year ASCVD risk score level for miners in imputed dataset. Table S3: Associations between patterns and 10-year ICVD risk score level for miners in imputed dataset. [file 12889_2019_8070_MOESM1_ESM.doc]

Supplementary Tables

**Table S1** Demographic characteristics described based on four dietary patterns.

| Demographic Factors | Ground workers N (%) | | | | P value | Underground workers N (%) | | | | P value |
| --- | --- | --- | --- | --- | --- | --- | --- | --- | --- | --- |
|  | Healthy dietary pattern N=273  (22.2) | High-fat and salt dietary pattern  N=359  (29.2) | High-salt dietary pattern N=392  (31.9) | Refined grains dietary pattern N=204  (16.6) |  | Healthy dietary pattern N=333  (23.7) | Northern dietary pattern N=421  (29.9) | High-fat dietary pattern N=437  (31.1) | High-salt dietary pattern N=213  (15.2) |  |
| Age |  |  |  |  | <.0001 |  |  |  |  | <.0001 |
| 35-44 | 134(49.1) | 151(42.1) | 146(37.2) | 51(25.0) |  | 163(48.9) | 165(39.2) | 248(56.8) | 96(45.1) |  |
| 45-54 | 116(42.5) | 158(44.0) | 177(45.2) | 102(50.0) |  | 135(40.5) | 220(52.3) | 166(37.9) | 102(47.9) |  |
| ≥55 | 23(8.4) | 50(13.9) | 69(17.6) | 51(25.0) |  | 35(10.5) | 36(8.6) | 23(5.3) | 15(7.0) |  |
| Gender |  |  |  |  | <.0001 |  |  |  |  | 0.63 |
| male | 139(50.9) | 297(83.7) | 236(60.2) | 175(85.8) |  | 332(99.7) | 421(100.0) | 436(99.8) | 213(100) |  |
| female | 134(49.1) | 62(17.3) | 156(39.8) | 29(14.2) |  | 1(0.3) | 0(0.0) | 1(0.2) | 0(0.0) |  |
| Education level |  |  |  |  | <.0001 |  |  |  |  | <.0001 |
| Bachelor degree or above | 54(19.8) | 45(12.5) | 36(9.2) | 13(6.4) |  | 13(3.9) | 3(0.7) | 28(6.4) | 13(6.1) |  |
| Junior college and senior high school | 163(59.7) | 218(60.7) | 241(61.5) | 115(56.4) |  | 193(57.9) | 206(48.9) | 275(62.9) | 110(51.6) |  |
| Junior high school or below | 56(20.5) | 96(26.7) | 115(29.3) | 76(37.3) |  | 127(38.1) | 212(50.4) | 134(30.7) | 90(42.3) |  |
| Marital status |  |  |  |  | 0.0154 |  |  |  |  | 0.34 |
| Married | 266(97.4) | 355(98.9) | 375(95.7) | 202(99.0) |  | 322(96.7) | 414(98.3) | 430(98.4) | 209(98.1) |  |
| others | 7(2.6) | 4(1.1) | 17(4.3) | 2(0.9) |  | 11(3.3) | 7(1.7) | 7(1.6) | 4(1.9) |  |
| Monthly income (RMB) |  |  |  |  | <.0001 |  |  |  |  | 0.17 |
| ≤4000 | 92(33.7) | 166(46.2) | 158(40.3) | 97(48.0) |  | 48(14.4) | 49(11.6) | 47(10.8) | 33(15.5) |  |
| 4000-6000 | 70(25.6) | 105(29.2) | 140(35.7) | 66(32.4) |  | 164(49.3) | 247(58.7) | 239(54.7) | 112(52.6) |  |
| 6000-8000 | 64(23.4) | 63(17.6) | 64(16.3) | 30(14.7) |  | 94(28.2) | 103(24.5) | 112(25.6) | 50(23.5) |  |
| ≥8000 | 47(17.2) | 25(6.9) | 30(7.7) | 11(5.4) |  | 27(8.1) | 22(5.2) | 39(8.9) | 18(8.5) |  |
| Smoke |  |  |  |  | <.0001 |  |  |  |  | 0.0061 |
| No | 186(68.1) | 148(41.2) | 240(61.2) | 77(37.8) |  | 133(39.9) | 134(31.8) | 137(31.4) | 56(26.3) |  |
| Yes | 87(31.9) | 211(58.8) | 152(38.8) | 127(62.5) |  | 200(60.1) | 287(68.2) | 300(68.7) | 157(73.7) |  |
| Drink |  |  |  |  | <.0001 |  |  |  |  | <.0001 |
| No | 207(75.8) | 175(48.8) | 307(78.3) | 103(50.5) |  | 192(57.7) | 256(60.8) | 189(43.3) | 96(45.1) |  |
| Yes | 66(24.2) | 184(51.3) | 85(21.7) | 101(49.5) |  | 141(42.3) | 165(39.2) | 248(56.8) | 117(54.9) |  |
| BMI |  |  |  |  | 0.0003 |  |  |  |  | 0.53 |
| ≤23 | 90(32.9) | 78(21.7) | 114(29.1) | 51(25) |  | 113(33.9) | 121(28.7) | 117(26.8) | 65(30.5) |  |
| 23-27.5 | 139(50.9) | 180(50.1) | 183(46.7) | 110(53.9) |  | 153(45.9) | 212(50.4) | 223(51.0) | 106(49.8) |  |
| >27.5 | 44(16.1) | 101(28.1) | 95(24.2) | 43(21.1) |  | 67(20.1) | 88(20.9) | 97(22.2) | 42(19.7) |  |
| Blood Pressure |  |  |  |  | 0.08 |  |  |  |  | 0.27 |
| Normal | 91(33.3) | 93(25.9) | 110(28.1) | 55(26.9) |  | 87(26.1) | 117(27.8) | 113(25.9) | 49(23.0) |  |
| Elevated | 111(40.7) | 143(39.8) | 162(41.3) | 71(34.8) |  | 140(42.0) | 164(38.9) | 172(39.4) | 74(34.8) |  |
| Hypertension | 71(26.0) | 123(34.3) | 120(30.6) | 78(38.2) |  | 106(31.8) | 140(33.3) | 152(34.8) | 90(42.3) |  |
| Physical activity level |  |  |  |  | 0.82 |  |  |  |  | 0.0053 |
| Inactive | 6(2.2) | 9(2.5) | 8(2.0) | 6(2.9) |  | 8(2.4) | 1(0.2) | 5(1.14) | 7(3.29) |  |
| Minimally Active | 101(37.0) | 140(39.0) | 133(33.9) | 72(35.3) |  | 88(26.4) | 87(20.7) | 113(25.9) | 60(28.2) |  |
| Health-enhancing physical activity | 166(60.8`) | 210(58.5) | 251(64.0) | 126(61.8) |  | 237(71.2) | 333(79.1) | 319(73.0) | 146(68.5) |  |
| Family history |  |  |  |  | 0.17 |  |  |  |  | 0.0286 |
| No | 145(53.1) | 210(58.5) | 199(50.8) | 115(56.4) |  | 217(65.2) | 254(60.3) | 251(57.4) | 145(68.1) |  |
| Yes | 128(46.9) | 149(41.5) | 193(49.2) | 89(43.6) |  | 116(34.8) | 167(39.7) | 186(42.6) | 68(31.9) |  |

**Table S2** Associations between dietary patterns and 10-year ASCVD risk score level for miners in imputed dataset ^a^.

| ASCVD risk score level | Ground workers  N=1372 | | | | P value | Underground workers  N=1533 | | | | P value |
| --- | --- | --- | --- | --- | --- | --- | --- | --- | --- | --- |
|  | Healthy pattern | High-fat and salt pattern | High-salt pattern | Refined grains pattern |  | Healthy pattern | Northern  Pattern | High-fat pattern | High-salt pattern |  |
| N (%) | 319(23.2) | 374(27.2) | 437(31.8) | 242(17.6) |  | 366(23.9) | 514(33.5) | 431(28.1) | 222(14.5) |  |
| Model 1 ^b^ | 1.00 | 2.17  (1.57-3.03)  P<.0001 | 1.47  (1.06-2.04)  P=0.0216 | 2.77  (1.94-3.96)  P<.0001 | <.0001 | 1.00 | 0.93  (0.69-1.24)  P=0.636 | 1.14  (0.85-1.53)  P=0.373 | 1.55  (1.11-2.18)  P=0.0107 | 0.0120 |
| Model 2 ^b^ | 1.00 | 1.31  (0.92-1.85)  P=0.135 | 1.43  (1.01-2.04)  P=0.0441 | 1.83  (1.26-2.67)  P=0.0017 | 0.0177 | 1.00 | 0.94  (0.70-1.26)  P=0.675 | 1.08  (0.80-1.45)  P=0.627 | 1.50  (1.07-2.11)  P=0.0192 | 0.0307 |
| Model 3 ^b^ | 1.00 | 1.12  (0.79-1.60)  P=0.528 | 1.29  (0.90-1.84)  P=0.161 | 1.67  (1.14-2.44)  P=0.0091 | 0.0423 | 1.00 | 0.84  (0.63-1.13)  P=0.253 | 1.00  (0.74-1.35)  P=0.991 | 1.49  (1.06-2.09)  P=0.0236 | 0.0073 |

^a^10-year ASCVD risk score level, 10-year atherosclerosis cardiovascular diseases risk score level; Imputed dataset, the dataset filled the missing data of the dietary intake (N=2906).

^b^ Model 1 included the dietary patterns. Model 2 adjusted for sex and drinking status. Model 3 adds education level, monthly income, marital status, BMI, physical activity level and family history to the above.

**Table** **S3** Associations between dietary patterns and 10-year ICVD risk score level for miners in imputed dataset ^a^.

| ICVD risk score level | Ground workers  N=1373 | | | | P value | Underground workers  N=1533 | | | | P value |
| --- | --- | --- | --- | --- | --- | --- | --- | --- | --- | --- |
|  | Healthy pattern | High-fat and salt pattern | High-salt pattern | Refined grains pattern |  | Healthy pattern | Northern  Pattern | High-fat pattern | High-salt pattern |  |
| N (%) | 319(23.2) | 374(27.2) | 437(31.8) | 243(17.7) |  | 366(23.9) | 514(33.5) | 431(28.1) | 222(14.5) |  |
| Model 1 ^b^ | 1.00 | 2.31  (1.46-3.66)  P=0.0004 | 1.77  (1.12-2.81)  P=0.0155 | 3.06  (1.89-4.95)  P<.0001 | <.0001 | 1.00 | 1.07  (0.71-1.61)  P=0.759 | 1.63  (1.09-2.44)  P=0.0167 | 1.92  (1.22-3.03)  P=0.0049 | 0.0036 |
| Model 2 ^b^ | 1.00 | 1.53  (0.95-2.46)  P=0.078 | 1.78  (1.11-2.86)  P=0.0176 | 2.17  (1.32-3.56)  P=0.0022 | 0.0166 | 1.00 | 1.08  (0.71-1.63)  P=0.721 | 1.52  (1.01-2.28)  P=0.045 | 1.84  (1.16-2.90)  P=0.0092 | 0.0166 |
| Model 3 ^b^ | 1.00 | 1.39  (0.87-2.25)  P=0.171 | 1.63  (1.01-2.62)  P=0.0470 | 1.93  (1.17-3.19)  P=0.0099 | 0.059 | 1.00 | 0.99  (0.65-1.50)  P=0.954 | 1.44  (0.95-2.17)  P=0.083 | 1.83  (1.15-2.91)  P=0.0106 | 0.0096 |

^a^ 10-year ICVD risk score level, 10-year ischemic cardiovascular diseases risk score level; Imputed dataset, the dataset filled the missing data of the dietary intake (N=2906).

^b^ Model 1 included the dietary patterns. Model 2 adjusted for sex and drinking status. Model 3 adds education level, monthly income, marital status, physical activity level and family history to the above.
